# Supplementary figures and images for: Performance of a rapid diagnostic test for the detection of Cryptosporidium spp. in African children admitted to hospital with diarrhea
Source: PLoS Negl Trop Dis. 2020 Jul 13;14(7):e0008448. doi: 10.1371/journal.pntd.0008448 (PMC7377516; doi:10.1371/journal.pntd.0008448)

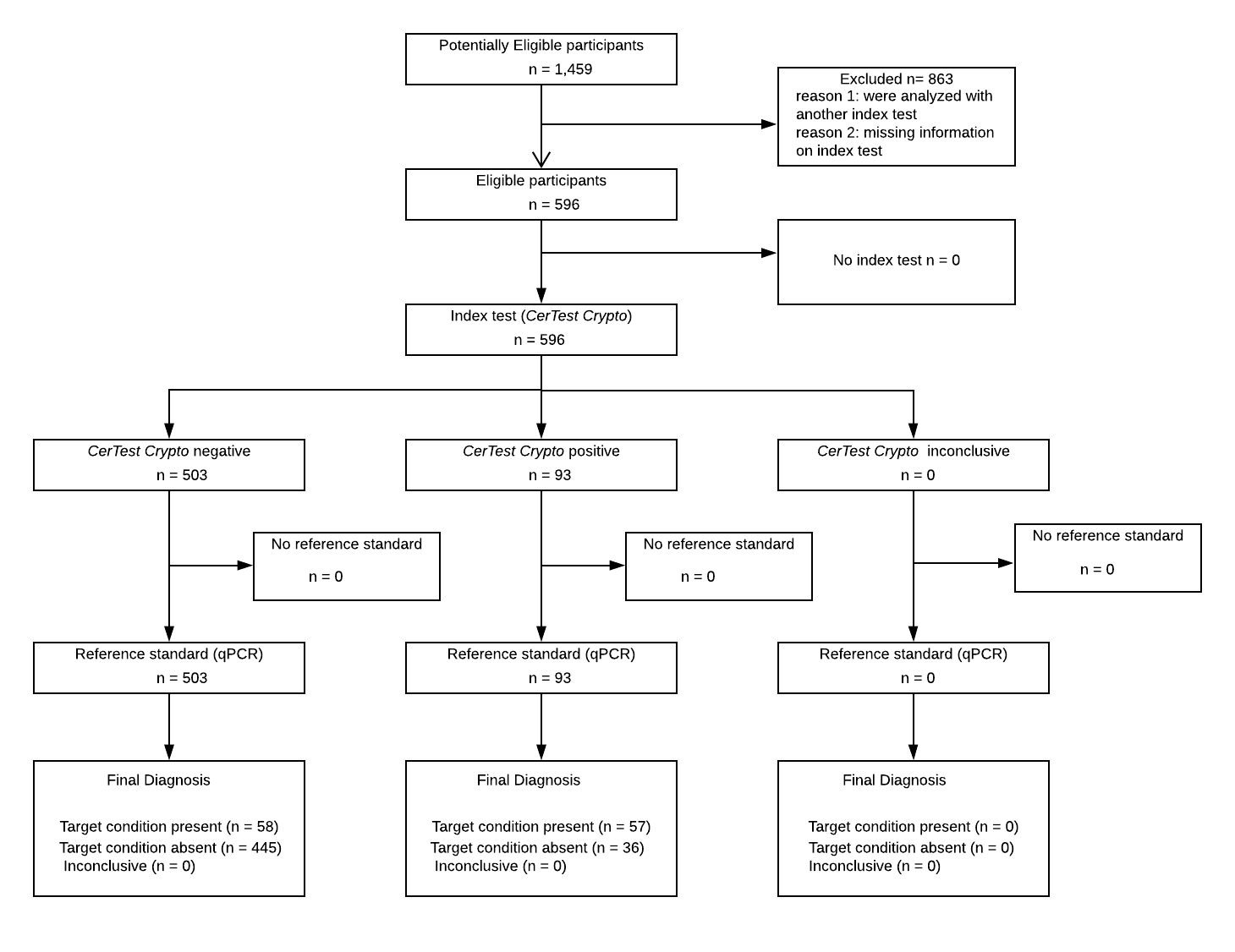

Supplement: S1 Diagram — (PNG) [file pntd.0008448.s002.png]

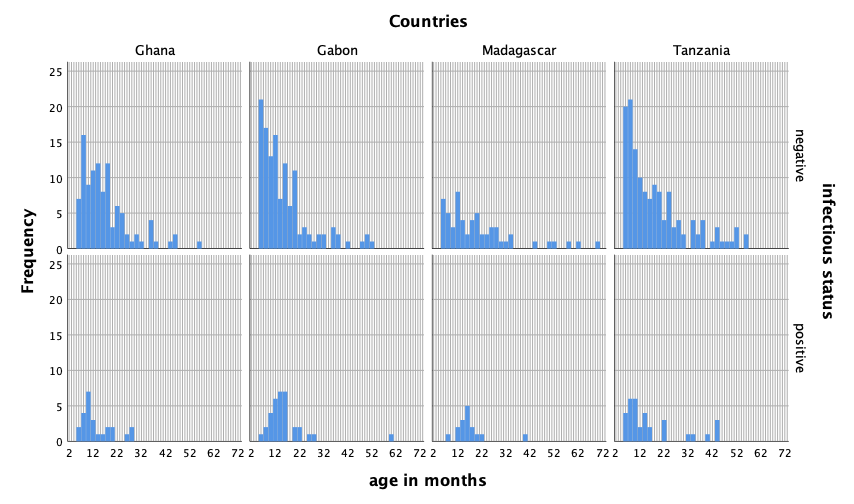


Figure S1: Distribution of infected stools across sites and age in months

Supplement: S1 Fig — (DOCX) [file pntd.0008448.s006.docx]
