# Supplementary material for: Performance of a rapid diagnostic test for the detection of Cryptosporidium spp. in African children admitted to hospital with diarrhea
Source: PLoS Negl Trop Dis. 2020 Jul 13;14(7):e0008448. doi: 10.1371/journal.pntd.0008448 (PMC7377516; doi:10.1371/journal.pntd.0008448)
Supplement: S1 Table — (DOCX) [file pntd.0008448.s004.docx]

Supplementary Table S4: Proportion of *Cryptosporidium*-PCR positives and demographic data across the four study sites

| **Country** | **Ghana (N=132)** | **Gabon**  **(N=192)** | **Madagascar**  **(N=83)** | **Tanzania**  **(N=189)** | **P-value** |  |
| --- | --- | --- | --- | --- | --- | --- |
| **Sex [n/N (%)]**  **Female**  **Male** | 12/132 (9)  13/132 (9.8) | 19/192 (9.9)  19/192 (9.9) | 10/83 (12)  8/83 (9.6) | 17/189 (9)  16/189 (8.5) | 0.87  0.96 | |
| **Age group [n/N (%)]**  **≤ 2 years**  **˃ 2 years** | 25/132 (18.9)  0/132 (0) | 37/192 (19.3)  1/192 (0.5) | 17/83 (20.5)  1/83 (1.2) | 29/189 (15.3)  4/189 (2.1) | 0.68  0.24 | |
| **Sampling period** | 05-2017 –  04-2018 | 05-2017 –  04-2018 | 05-2017 –  04-2018 | 05-2017 –  04-2018 | - | |
| **Average rainfall (mm)** | 120.7 | 166.6 | 121.3 | 107.5 | - | |

**n**: *Cryptosporidium*-PCR positive cases

**P-value** for testing difference between demographic data (Sex and Age) and all four study sites
