# Supplementary material for: Performance of a rapid diagnostic test for the detection of Cryptosporidium spp. in African children admitted to hospital with diarrhea
Source: PLoS Negl Trop Dis. 2020 Jul 13;14(7):e0008448. doi: 10.1371/journal.pntd.0008448 (PMC7377516; doi:10.1371/journal.pntd.0008448)
Supplement: S2 Table — (DOCX) [file pntd.0008448.s005.docx]

S2 Table: Proportion of *Cryptosporidium*-PCR positives and demographic data across the four study sites

|  | | N(%) |  | Positive (n) /N (%) | | | |
| --- | --- | --- | --- | --- | --- | --- | --- |
|  |  |  | **Total** | **Ghana** | **Gabon** | **Madagascar** | **Tanzania** |
| All | |  | **115/596** | 25/132(18.9) | 38/192(19.8) | 18/83(21.7) | 34/189(18.0) |
| Sex | |  |  |  |  |  |  |
|  | Female | 271(45.5) | 58/271(21.4) | 12/58 (20.7) | 19/79(16.8) | 10/42 (19.5) | 17/92(18.5) |
|  | Male | 325(54.5) | 57/325(17.5) | 13/74 (17.6) | 19/113 (24.1) | 8/41 (23.8) | 17/97 (17.5) |
|  | P-value |  | 0.23 | 0.65 | 0.14 | 0.42 | 0.50 |
| Age in month  (mean ±SD) | | 15.2 ± 11.6 | 14.45±8.9 | 13.6 ± 9.5 | 13.0 ± 10.3 | 17.5 ± 13.7 | 16.3 ± 12.7 |
| Age class,  in month | |  |  |  |  |  |  |
|  | 0-6 | 123(20.6) | 14/123(11.4) | 2/12(16.7) | 5/55(09.1) | 2/16 (12.5) | 5/40 (12.5) |
|  | 6-12 | 186(31.2) | 43/186(23.1) | 14/50(28.0) | 12/58(20.7) | 3/19 (15.8) | 14/59 (23.7) |
|  | 12-18 | 125(21.0) | 34/125(27.2) | 4/36(11.1) | 14/39(35.9) | 10/20 (50.0) | 6/30 (20.0) |
|  | 18-24 | 70((11.7) | 11/70(15.7) | 2/16(12.5) | 4/20(20.0) | 2/11(18.2) | 3/23(13.0) |
|  | 24-30 | 31(05.2) | 5/31(16.1) | 3/8(37.5) | 2/7(28.6) | 0/7(0.0) | 0/9(0.0) |
|  | 30-36 | 21(03.5) | 2/21(09.5) | 0/5 (0) | 0/5(00.0) | 0/3 (0.0) | 2/8 (25.0) |
|  | >36 | 40(06.7) | 6/40(15.0) | 0/5(0) | 1/8(12.5) | 1/7(14.3 | 4/20(20.0) |
|  | P-value |  | **0.023** | 0.11 | **0.04** | **0.02** | 0.37 |
|  |  |  |  |  |  |  |  |
| Sampling period | | - |  | 05-2017 – 04-2018 | 05-2017 – 04-2018 | 05-2017 – 04-2018 | 05-2017 – 04-2018 |
| Average rainfall (mm) | | - |  | 120.7 | 166.6 | 121.3 | 107.5 |

**n**: *Cryptosporidium*-PCR positive cases

N: Total

**P-value** for testing difference between demographic data (Sex and Age) and all four study sites
