# Supplementary material for: Performance of a rapid diagnostic test for the detection of Cryptosporidium spp. in African children admitted to hospital with diarrhea
Source: PLoS Negl Trop Dis. 2020 Jul 13;14(7):e0008448. doi: 10.1371/journal.pntd.0008448 (PMC7377516; doi:10.1371/journal.pntd.0008448)
Supplement: Attachment — Submitted filename: Rebutal letter_PLOS neglected Tropical Diseases.docx [file pntd.0008448.s008.docx]

November 26^th^, 2019.

To the Editor,

PLOS Neglected Tropical Disease.

Letter in response to reviewer comments on the PLOS Neglected Tropical Disease submission (PNTD-D-19-01374)

Project Title: **Performance of a rapid diagnostic test for the detection of *Cryptosporidium* spp. in African children admitted to hospital with diarrhea.**

Dear Editor,

On November 13^th^, 2019, we received a decision on our PLOS Neglected Tropical Disease submission (PNTD-D-19-01374), whereby the reviewers appreciated the attention to an important problem on the diagnosis of cryptosporidiosis using a rapid diagnostic test, but raised some substantial concerns about the current state of our manuscript. In response to the reviewer comments we have made important readjustments to address key issues concerning our methodology and result interpretation. The following text highlights key changes that have been made to the manuscript to address the reviewer concerns.

**Section 1: comments related to methodology**

**Reviewer 1 comment 1**: “They didn’t mention anything regarding storage of their stool samples for CerTest Crypto. What was the timing of the test after collecting the stool samples? Did the test run on the same day of collecting stool samples. More details need to be given about this CerTest. From where they procured this test”.

**In response to this comment,** a clarification has been made in the section on materials and methods subtitled “**CerTest Crypto RDT processing”** Lines148-152:

**Change:** Fresh stool samples were tested using a commercially available RDT (CerTest BIOTEC S.L, Pol. Industial Rio Gallego II, Zaragoza Spain). The RDTs were stored at room temperature in all the sites according to manufacturer instructions. The stool samples were collected and transported in cool boxes (4°C) to the laboratory. All samples were analysed within 24 hours of sample collection. Stool sample aliquots were stored at -80°C for further analysis

**Reviewer 1 comment 2:** “The primer-probe set used in this study was specific for C. Parvum and not for Cryptosporidium species”.

**In response to this comment:** The primers and probes are indeed specific for *Cryptosporidium parvum*. However, they can also detect other *Cryptosporidium* species including *C. hominis*. How good they are to detect other species is not very well evaluated. Our reference mentions only *C. parvum* as previously *C. hominis* was categorized under *C. parvum*. Furthermore, RFLP PCR was performed to detect all *Cryptosporidium* species. Therefore, we don’t think we missed any important species diagnosis, or we were biased towards any specific species. An additional line with clarifications has been added in the section on materials and methods: Real time PCR.  **‘**Lines182-185’

**Change:** No change

**Reviewer 2 comment 1:**

“Ethical approval: Some important details are needed about the lab tech training both on CerTest Crypto RDT using and PCR test (one training including the lab tech of all sites? Or one training per site? The training was followed by trainer’s assessment or not?). No mention of quality control and quality assurance in the materials and methods part. Why? Kindly clarify”.

**In response to this comment**, additional information is included in the sections on “Field and laboratory procedures , Lines 138-145” and “CerTest Crypto RDT processing Lines 157-164”, to address issues regarding training on use of Crypto RDT, qPCR and quality control quality assurance measures.
**Change:** Lines 138-145:Prior to the start of the study, a general training was organized in Tanzania with participants from all the four study sites in attendance. Training was based on CerTest Crypto RDT testing and qPCR for Cryptosporidium: Lab technicians from all the four sites were trained on Sample processing, time of testing, and protocols were developed and harmonized for use in all the four sites. The Standard Operation Pocedures (SOPs) for sample processing and testing with the CerTest Crypto RDT and qPCR were implemented in all the four sites.

Lines 157-164: The test results were not read later than 10 minutes as instructed by manufacturer (11). The Certest Crypto Kit are supplied with quality control reagents. The quality control reagent is a known positive and negative sample. One negative and one positive control was run once a month in all the four sites and for each new batch of test kits. In addition, an internal procedural control included in the test, wherein the appearance of a green line in the control line result window is an indication of correct procedural technique and confirms enough specimen volume was used.

**Reviewer 2 comment 2:**

Lines 114-116: “All hospitals where participants were recruited are situated in semi-urban areas across all study sites. All samples collected within this study were used to evaluate the test performance of CerTest Crypto compared to the composite reference standard of qPCR and RFLP-PCR”. Please, give provide clarifications (or rephrase) since this is not observed in the results (cf Tables 1 and 2).

**In response to this comment:** First of all, the idea was not to highlight the results from each PCR separately but to combine results from all PCR conducted (qPCR, RFLP-PCR) positive if either method yielded a positive result, otherwise negative. That’s why we did not provide different tables with the results of each PCR. The mentioned the sentence “Samples used to evaluate the test performance of CerTest Crypto compared to the composite reference standard of qPCR and RFLP-PCR are collected from these health centers” to remind somehow the main objective of our study.

The second sentence of the commented section was revised.

**Change:** Lines 128-130: Samples used to evaluate the test performance of CerTest Crypto compared to the composite reference standard of qPCR and RFLP-PCR are collected from these health centers.

**Reviewer 2 comment 3**

Line 122: “As indicated in Ethical approval it is better to write: The parents or legal guardian of the patient who provided the signed informed consent …..”

**In response to this comment,** the text was replaced in the section “**Field and Laboratory procedures**

“ Line 136

**Change:** “The parents or legal guardian of the patient who provided the signed informed consent”

**Reviewer 2 comment 4**

Line 180: “Please add: of Ghana (the Committee On Human Research, Publications And Ethics, Kwame Nkrumah University Of Science And Technology of Ghana, …)”

**In response to this comment**, the text was amended to section “**Ethical approval** “ Line 222

**Change:** “of Ghana” was added after …..Kwame Nkrumah University of Science and Technology

**Section 2: comments related to results**

Reviewer 1 comment 1:

“The result is poorly written; the quality of experimental data needs to be improved. Frequency distribution of RDT test results in Table 1.

**In response to this comment**: It is not entirely clear to the authors what the reviewer is suggesting to change in Table1. We changed the title of the table to make is clearer that RTD results are presented. The result section of the manuscript has been reviewed to make it more readable.

**Change**: No change

Presentation of Table 2 and Table 3 are not very clear. The authors are requested to compare. Sensitivity, Specificity, Positive Predictive Value (PPV) and, Negative Predictive Value (NPV) of RDT when compared with qPCR assay should be mentioned”.

**In response to this comment**: Changes made to the titles of tables 1 and 2 are highlighted.

**Change**: **Table 2.** Overall and country specific test evaluation results of the RDT when compared to qPCR and RFLP as reference standard.

**Table 3.** Overall and country specific test evaluation results of the RDT when compared to qPCR and RFLP as reference standard, excluding test results of *Cryptosporidium* meleagridis“, “*Cryptosporidium* xiaoi/bovis“.

Reviewer 2:

Comment 1

“The analysis presented match the analysis plan, However many complementary information will need to clarify some points”.
Lines 185-188: “Please, remove this sentence here and completed materials and methods with: To ensure a comparable ….. to April 2018”.

**In response to the above comment**: At the beginning of the main project, all four sites were not using the same rapid diagnostic test. For example, Ghana was using the Quick-Rapid test in the beginning. Therefore, to ensure comparability, we considered for analysis all RDT results based on the CerTest.

**Change**: No change

Comment 2
Line 192: “Please, replace all 4 sites by the four sites”.

**In response to the above comment,**  all 4 sites have been replaced with “the four sites” throughout the manuscript as suggested.

**Change**: four sites was added in Lines 141-142;145;161;233; 261;309;320

Comment 3
Lines 196-200: Please remove “These performance characteristics …were excluded from the analysis”.  Better in materials and methods part.

**In response to the above comment**: the text referred in lines 208-212 has been rephrased but not moved from the section on Statistical analysis.

**Change:** the text has been rephrased

**Section 3: Comments related to Discussion**

“Your study shows intra sites sensitivity compared to Rapid test specificity (The sensitivity of the test varied considerably in all study sites being highest in Madagascar (72.22%) in comparison to Gabon and Ghana which had 50 and 52 % respectively and was lowest in Tanzania with 35.29 %). Kindly, give some eventual raisons (training? cerTest Crypto RDT conservation? cerTest Crypto RDT manufacturing? samples conservation????)”

**In response to this comment**, we don’t have another explanation, except the one given in a discussion section, meaning “…… several other factors including low parasite densities as reported elsewhere”. Also, the same procedures and QC/QA were applied at the four site sites, given a little room for quality concerns.
**Change:** No change
**Limitations**
“The absence of microscopic data is a real limitation of these results”

**In response to the above comment**, our limitations that are mainly due to absence of microscopic data has been discussed in the manuscript

**Change**: No change
“The absence of QC and QA will need to be discussed if it’s the case”

**In response to the above comment,** we have mentioned the quality control aspect in the section on field and laboratory procedures and CerTest Crypto RDT processing. The quality control of test kit was performed once each month and for each new batch of test kits in all the four sites. Therefore, we do not think this can be a limitation of the performance of the test kit

**Change**: No change

“References
Please, updated the references. For example, the references below should be helpful
Bouzid et al, Clin Microbiol Rev. 2013: 115–134. doi: 10.1128/CMR.00076-12
Garcia et al, Clin Microbiol Rev. 2018: e00025-17. doi: 10.1128/CMR.00025-17
Cunha et al, Rev Inst Med Trop Sao Paulo. 2019; 61: e28. doi: 10.1590/S1678 9946201961028
Sylvia Afriyie Squire, Una Ryan, Parasit Vectors. 2017; 10: 195. doi: 10.1186/s13071-017-2111-y
Moore et al, PLoS Negl Trop Dis. 2016 Jul; 10(7): e0004822. Published online 2016 Jul 7. doi: 10.1371/journal.pntd.0004822”

**In response to the above comment**, we have included some of the suggested references in the introduction section of the manuscript. Changes and references are highlighted

**Change:** Lines 75-77, 78-80 and 91-92

**Section 4: Comments related to Conclusion**

Reviewer 1: “Conclusion is not very clear by the data presented”.

**The response to the above comment:** The conclusion has been rephrased

**Change:** Therefore, additional research is needed to evaluate the performance of the CerTest Crypto RDT with particular emphasis on light intensity infections whereby the concentrations of antigens present in the sample are below the detection limit of the test that may result in an increase number of false negatives

Reviewer 2:

“The conclusions of this study supported the data presented but the limitations of analysis didn't clearly describe”.

“The authors discuss how these data can be helpful to advance our understanding of the topic under study and the public health relevance was addressed”

**The response to the above comments:** We thank the reviewer for their comments

**Change**: No change

**Section 5: Editorial and Data Presentation Modifications**

Reviewer #1: Many changes     will be required

**The response to the above comment:** We agree

Reviewer #2: No comment

Reviewer #3: See "Summary and General Comments Section" for detailed comments

**Section 6: Summary and General Comments**

Reviewer #1: This is paper of interest but, poorly written and need to be revised

**The response to the above comment**: Revision was made

**Change**: See the manuscript

Reviewer #2:

“Most human infections causing diarrhea are attributed to two species: Crytosporidium parvum (zoonotic transmission) and C. hominis (anthroponotic transmission). Overall this is an important paper for improving epidemiology and diagnostic of Cryptosporidium parasites using CerTest Crypto rapid diagnostic test; therefore could lead to better and faster management of diarrhea cases in our hospitals.
I have a few general comments. Please add in the introduction the background of the different cryptosporidium species in the four countries and clearly update diagnostic tests validated by the WHO to date. Although well readable, the text would benefit from a review by a professional editor include as co-authorships. The English is not fluent in several passages and it looks like the text has been translated from French”.

**The response to the above comment:** As you might know Cryptosporidium joined the Neglected Disease initiative in 2006 because some important questions are raised on the knowledge of this pathogen. There is still many information lacking on Cryptosporidium worldwide, thus the main project which is the umbrella of our study will provide the first information on the diversity of Cryptosporidium for some countries such us Gabon. Therefore we cannot provide any information related Cryptosporidium species in the four countries as requested. There is also no recommendation by WHO in terms of RDT for Cryptosporidium detection, even the gold standard method is not yet established.

**Change**: No change

Reviewer #3: Summary statement: The authors presents an analysis of the efficacy of a novel Cryptosporidium rapid diagnostic test. The manuscript is clear and addresses an important concern – specifically, that RDTs may be efficacious in the lab but their reliability and utility in the field is more important. The authors qualitatively evaluate the potential ‘batch effect’ variability in the assay; however, relevant details about the study and study design should be included.

There may be a demographic bias rather than batch bias of the test. For example, the assay may be more efficacious when testing in young infant (i.e. using age as a proxy for parasitemia), vs. children and adults. Alternatively, it might be more effective in children who are at low risk for multiple infections rather than high risk for multiple infections (i.e. addressing the author’s concern about off target organisms). By presenting more of the study design and cohort demographic data, these considerations could be evaluated.
**The response to the above comment** We thank the reviewer for his comments, indeed only children under five are included at the four study sites as indicated at the MM section.

**Change**: No change

**Major concerns:**
1) “Is this the first report of this cross-sectional study (or the cohort represented in the study)? If so, greater methodological detail is necessary. If not, please include a citation”

**In response to the above concern**, this is the first report of this cross-sectional study.

**Change:** See material and method section

2) “Please include an analysis of patient demographics, per my point in the summary statement”.

**In response to the above comment**, we have stated in the manuscript that this study is a sub-study of a larger cross-sectional study that aimed to determine the zoonotic or anthroponotic C. parvum and C. hominis subspecies in children under five with diarrhea, in close contacts of infected children and in water and animal sources in order to delineate transmission routes. Thus, we don’t see a potential scientific added value of patient demographic.

**Change**: No change

3) “Please clarify in the ‘Real-time PCR’ section which species could be detected with these pan-Crypto primers”.

**In response to the above comment**, we have stated in the section on Real-time PCR of the manuscript which species are detected by the crypto primers used in our study

**Change**: No change

4) “Table 1 – what do the dashes mean? If 0, please replace with 0”

**In response to the above concern** the dashes mean 0 and these have replaced on the table

**Change**:
5) “Is the protein targeted in the CerTest assay known? Can it be BLAST-ed to identify which organisms might be responsible for the hypothesized cross-reaction from other protozoa?”

**In response to the above concern**, the name of the protein target in the CerTest assay is not provided by the manufacturer

**Change**: No change
6) “On lines 96-98, the authors mention that RDTs tend to have reduced sensitivity with uncommon species. What species are defined as ‘uncommon’? Naming the species would make it clear if this problem motivated Table 2”

**Response to the above comment** Change were made at line lines 108-109

**Change**: ”In addition, these RDTs demonstrate varying performances, and some of them are not well validated for the detection of less frequent non-parvum/hominis Cryptosporidium species.

7) “Line 308 – without knowing more about the RDT, the following statement is not clear: ‘observed color intensity on the test window in case of light infections. It sounds as if the authors are suggesting that the intensity of the test color band could be difficult to see in light infections. The manufacturer’s instructions available online do not include any text related to interpreting presence/absence of the band in cases where intensity is questionable. The authors should update the methods section to include details of the training provided to field workers related to interpretation of the CerTest results”.

**The response to the above comment** has been addressed in the concluding remarks of the manuscript. lines 349-353

**Change**: “Therefore, additional research is needed to evaluate the performance of the CerTest Crypto RDT with particular emphasis on light intensity infections whereby the concentrations of antigens present in the sample are below the detection limit of the test that may result in an increase number of false negatives”

**Minor concerns:**

1) “Please add references for claims about the assay (Around line 100)”

**In response to the above concern** a reference has been added in line 112

**Change**:
2) “Line 133 – Please provide a reference for the manufacturer’s instructions”.

**In response to the above concern** a reference has been added in line 158
**Change**:
3) “Line 135 – ‘modified MO BIO-Qiagen stool DNA extraction protocol’ Were both the MO-BIO and Qiagen versions of the kit used, and if so could there be an extraction bias with the different versions?:

**In response to the above concern**, we started with MO BIO PowerSoil DNA Isolation kit that was formerly sold by MO BIO to Qiagen and became so far PowerSoil DNA Isolation Kit-Qiagen. Only the seller was changed and not the manufacturer

**Change:** No change

**Grammar/copy edit suggestions:**
“Lines 71-73: ‘Cryptosporidiosis which is caused by a coccidian parasite belonging to the genus Cryptosporidium has been recognized as one of the major causes of diarrheal disease worldwide (2).’ – this is grammatically incorrect. I’d suggest replacing it with ‘Cryptosporidiosis is caused by a coccidian parasite belonging to the genus Cryptosporidium and has been recognized as one of the major causes of diarrheal disease worldwide”

**In response to the above concern** the suggested change has been made and the text replaced

**Change**: Cryptosporidiosis is caused by a coccidian parasite belonging to the genus Cryptosporidium and has been recognized as one of the major causes of diarrheal disease worldwide (2)” is added in lines 73-75

“Lines 82-84: ‘All of these methods have some drawbacks that include being time-consuming and relatively expensive, require well-equipped laboratories and well-trained or skilled personnel’ – Inappropriate use of semi colon. I’d suggest replacing this text with ‘All of these methods have drawbacks including being time-consuming and relatively expensive, as well as requiring well-equipped laboratories and well-trained or skilled personnel”

**In response to the above concern** the suggested change has been made and the text replaced

**Change**: “All of these methods have drawbacks including being time-consuming and relatively expensive, as well as requiring well-equipped laboratories and well-trained or skilled personnel” has been added in lines 93-95

“line 98 – Please add new paragraph character”

**In response to the above concern**: we agree the new paragraph starts now at line 111

**Change:** the suggested change has been made
“Line 125: define SOP”

**In response to the above concern** a definition of SOP has been provided

**Change:** Standard Operation Procedures (SOPs) was added in Line 142
“Line 136 – ‘Inhibitors’ should be ‘inhibitors”

**In response to the above concern** the suggested change has been made

**Change:** inhibitors is added in Line 168

A number of abbreviations are presented that are unnecessary (i.e. not ever used again).
Cryptosporidium is periodically not italicized”

**In response to the above concern** the suggested change has been

**Change:** Cryptosporidium is italicized throughout the manuscript
“Line 304 – ‘species should be ‘species’

**In response to the above concern** the two words suggested here are similar.

**Change**: No change

While we hope we have substantially addressed the reviewer concerns, we look forward to a favorable response and will be ready to provide any further clarifications should need be

Respectfully
