# Supplementary material for: Performance of a rapid diagnostic test for the detection of Cryptosporidium spp. in African children admitted to hospital with diarrhea
Source: PLoS Negl Trop Dis. 2020 Jul 13;14(7):e0008448. doi: 10.1371/journal.pntd.0008448 (PMC7377516; doi:10.1371/journal.pntd.0008448)
Supplement: Attachment — Submitted filename: Reviewercomment.docx [file pntd.0008448.s009.docx]

Reviewer comment

Authors described clearly the study methods with objectives also clearly articulated. Study population is well described with adequate sample size power and the conclusions were supported by correct statistical analysis. In this study all analysis match the analysis plan and the results are presented accordingly with good tables and figures. In this reviewed version, the conclusions are supported by data presented and authors have discussed how data can be helpful to advance the study topic understanding. However some limitations will need to be clarify (ex. microscopic data). In conclusion, this manuscript can be accepted for publication in PloS NTD.
