# Supplementary material for: Performance of a rapid diagnostic test for the detection of Cryptosporidium spp. in African children admitted to hospital with diarrhea
Source: PLoS Negl Trop Dis. 2020 Jul 13;14(7):e0008448. doi: 10.1371/journal.pntd.0008448 (PMC7377516; doi:10.1371/journal.pntd.0008448)
Supplement: Attachment — Submitted filename: Rebuttal letter_PLOS neglected Tropical Diseases_MM.docx [file pntd.0008448.s010.docx]

March 8^th^, 2020.

To the Editor,

PLOS Neglected Tropical Disease.

Letter in response to reviewer comments on the PLOS Neglected Tropical Disease submission (PNTD-D-19-01374)

Project Title: **Performance of a rapid diagnostic test for the detection of *Cryptosporidium* spp. in African children admitted to hospital with diarrhea.**

Dear Editor,

On February 20^th^, 2020, we received a decision on our PLOS Neglected Tropical Disease submission (PNTD-D-19-01374), whereby the reviewers find the scientific content of our manuscript acceptable. However, the reviewers have suggested some clarifications on the limitations of the study, especially on the demographic effects of the test performance. Following the reviewer recommendations, we have provided some clarity on the limitation due to the absence of microscopy data. We also included an additional table as supplementary document emphasizing demographic data across geographic areas. The following text highlights key changes that we made to the manuscript to address the reviewer concerns.

**Limitations**
Reviewer #2:

“In this reviewed version, the conclusions are supported by data presented and authors discuss how data can be helpful to advance the study topic understanding. However, some limitations will need to be clarify (ex. microscopic data)”

**In response to the above comment**, we have provided further clarifications on the limitation due to the absence of microscopy data

**Change**: explanations were added on line 336 to 339

**Additional comment on demographic summaries**

Reviewer#3:

“We recommend including demographic summaries across sites for factors known to be associated with incidence of crypto (gender balance, average age at sampling, average monthly rainfall at each site”

**In response to the above comment**, the demographic data is summarized in a supplementary table (S5 Table). Indeed, we did not observe any geographical variability of Cryptosporidium infection according to the demographic characteristics across the four study sites. The trend of the distribution of *Cryptosporidium* spp was similar in all countries. Therefore, the demographic data collected from this study do not provide substantial evidence to argue for batch effect on the performance of the Certest Crypto. However, the younger aged tend to be associated with infection throughout the four study sites.

**Change**: was made at “Results section” on lines 255-257. Further comment was added in the “Discussion section” on line 303 to 306. Finally, an additional supplementary table has been added (see line 355-356)

While we hope we have substantially addressed the reviewer comments, we look forward to a favorable response and will be ready to provide any further clarifications should need be

Respectfully
