# Supplementary material for: Performance of a rapid diagnostic test for the detection of Cryptosporidium spp. in African children admitted to hospital with diarrhea
Source: PLoS Negl Trop Dis. 2020 Jul 13;14(7):e0008448. doi: 10.1371/journal.pntd.0008448 (PMC7377516; doi:10.1371/journal.pntd.0008448)
Supplement: Attachment — Submitted filename: Rebutal letter_PLOS neglected Tropical Diseases.docx [file pntd.0008448.s012.docx]

March 30^th^, 2020.

To the Editor,

PLOS Neglected Tropical Disease.

Letter in response to reviewer comments on the PLOS Neglected Tropical Disease submission (PNTD-D-19-01374)

Project Title: **Performance of a rapid diagnostic test for the detection of *Cryptosporidium* spp. in African children admitted to hospital with diarrhea.**

Dear Editor,

On March 24^th^, 2020, we received a decision on our PLOS Neglected Tropical Disease submission (PNTD-D-19-01374), whereby the reviewers find the scientific content of our manuscript acceptable. However, the reviewers have suggested that we make clarifications on the limitations of the study, especially on the demographic effects of the test performance. Following the reviewer recommendations, we have provided some clarity on the demographic data in a supplementary table with emphasis on the age distribution from all four study sites. The following text highlights key changes that we made to the manuscript to address the reviewer concerns.

**Limitations**
Reviewer #3:

“Demographic information as reported in the new Supplementary Table S4 are inconsistent. Based on sex only being indicated for ~10% of each population and age for less than ~20% for each population, it appears the authors have only indicated demographic information for the crypto-positive children. Demographic information from all tested children is necessary to determine whether differences in age or sex across cohorts influenced test performance. The authors should indicate the total number of male and female subjects within each site, not just the number of males and females that were PCR-positive.  A similar breakdown should be included for age. Additionally, a simple histogram with ~2-month resolution would easily demonstrate whether there are substantial differences in the distribution of subject age in each cohort.”

**In response to the above comment**, we provide in supplementary table S5 the distribution of infections according to the demographic characteristics for the four study sites. We further show a supplementary Figure S6 an histogram for the frequency of infections by age in month for samples from all four study sites. The frequency table suggest that younger children aged around 6 to 24 months tend to be associated with infection throughout the four study sites.

While we hope we have substantially addressed the reviewer comments, we look forward to a favorable response and will be ready to provide any further clarifications should need be

Respectfully
