# Supplementary material for: Performance of a rapid diagnostic test for the detection of Cryptosporidium spp. in African children admitted to hospital with diarrhea
Source: PLoS Negl Trop Dis. 2020 Jul 13;14(7):e0008448. doi: 10.1371/journal.pntd.0008448 (PMC7377516; doi:10.1371/journal.pntd.0008448)
Supplement: Attachment — Submitted filename: Reviewer Comment.docx [file pntd.0008448.s007.docx]

**Ref: PNTD-D-19-01374**

**Title: Performance of a rapid diagnostic test for the detection of Cryptosporidium spp. In African children admitted to hospital with diarrhea
Journal: Plos NTD**

Comments from the reviewers:

Most human infections causing diarrhea are attributed to two species: *Crytosporidium parvum* (zoonotic transmission) and *C. hominis* (anthroponotic transmission). Overall this is an important paper for improving epidemiology and diagnostic of *Cryptosporidium* parasites using CerTest Crypto rapid diagnostic test; therefore could lead to better and faster management of diarrhea cases in our hospitals.

I have a few general comments. Please add in the introduction the background of the different *cryptosporidium* species in the four countries and clearly update diagnostic tests validated by the WHO to date. Although well readable, the text would benefit from a review by a professional editors include as co-authorships. The English is not fluent in several passages and it looks like the text has been translated from French.

Some comments/suggested changes

**Abstract**

Lines 42, 43, 44 and throughout the manuscript: Please give harmonization in the confidence interval writing. For example you can use: ??,??% (95° CI: ??.??% - ??.??%)

**Materials and methods**

Some important details are needed about the lab tech training both on CerTest Crypto RDT using and PCR test (one training including the lab tech of all sites? Or one training per site? The training was followed by trainer’s assessment or not?).

No mention of quality control and quality assurance in the materials and methods part. Why? Kindly clarify.

Lines 114-116: “All hospitals where participants were recruited are situated in semi-urban areas across all study sites. All samples collected within this study were used to evaluate the test performance of CerTest Crypto compared to the composite reference standard of qPCR and RFLP-PCR”. Please, give provide clarifications (or rephrase) since this is not observed in the results (cf Tables 1 and 2).

Line 122: As indicated in Ethical approval it is better to write: The parents or legal guardian of the patient who provided the signed informed consent …..

**Ethical approval**

Line 180: Please add: of Ghana (the Committee On Human Research, Publications And Ethics, Kwame Nkrumah University Of Science And Technology of **Ghana**, …)

**Results**

Lines 185-188: Please, remove this sentence here and completed materials and methods with: To ensure a comparable ….. to April 2018.

Line 192: Please, replace all 4 sites by **the four** sites.

Lines 196-200: Please remove “These performance characteristics …were excluded from the analysis”. Better in materials and methods part.

**Discussion**

Your study shows intra sites sensitivity compared to Rapid test specificity (The sensitivity of the test varied considerably in all study sites being highest in Madagascar (72.22%) in comparison to Gabon and Ghana which had 50 and 52 % respectively and was lowest in Tanzania with 35.29 %). Kindly, give some eventual raisons (training? cerTest Crypto RDT conservation? cerTest Crypto RDT manufacturing? samples conservation????).

Limitations

The absence of microscopic data is a real limitations of these results

The absence of QC and QA will need to be discussed if it’s the case.

**References**

Please, updated the references. For example, the references below should be helpful

Bouzid et al, [Clin Microbiol Rev](https://www.ncbi.nlm.nih.gov/pmc/articles/PMC3553671/). 2013: 115–134. doi: [10.1128/CMR.00076-12](https://dx.doi.org/10.1128%2FCMR.00076-12)

Garcia et al, Clin Microbiol Rev. 2018: e00025-17. doi: 10.1128/CMR.00025-17

Cunha et al, Rev Inst Med Trop Sao Paulo. 2019; 61: e28. doi: 10.1590/S1678 9946201961028

Sylvia Afriyie Squire, Una Ryan, Parasit Vectors. 2017; 10: 195. doi: 10.1186/s13071-017-2111-y

Moore et al, PLoS Negl Trop Dis. 2016 Jul; 10(7): e0004822. Published online 2016 Jul 7. doi: 10.1371/journal.pntd.0004822

……………………..
